# Aneurysm Rupture Prediction Based on Strain Energy-CFD Modelling

**DOI:** 10.3390/bioengineering10101231

**Published:** 2023-10-21

**Authors:** Ahmed M. Al-Jumaily, Abd Halim Bin Embong, Mohammad AL-Rawi, Giri Mahadevan, Shukei Sugita

**Affiliations:** 1 Institute of Biomedical Technologies, Auckland University of Technology, Auckland 1010, New Zealand; 2 Mechatronics Department, Kulliyyah of Engineering, International Islamic University Malaysia, Kuala Lumpur 53100, Malaysia; ehalim@iium.edu.my; 3 Centre for Engineering and Industrial Design, Waikato Institute of Technology, Hamilton 3240, New Zealand; mohammad.al-rawi@wintec.ac.nz; 4 Department of General Surgery, Counties Manukau District Health Board, Auckland 1640, New Zealand; giri.mahadevan@middlemore.co.nz; 5 Centre for Fostering Young and Innovative Researchers, Nagoya Institute of Technology, Nagoya 466-8555, Japan; sugita.shukei@nitech.ac.jp

**Keywords:** CFD, aneurysm, energy strain function, mechanical properties, cyclic loading

## Abstract

This paper presents a Patient-Specific Aneurysm Model (PSAM) analyzed using Computational Fluid Dynamics (CFD). The PSAM combines the energy strain function and stress–strain relationship of the dilated vessel wall to predict the rupture of aneurysms. This predictive model is developed by analyzing ultrasound images acquired with a 6–9 MHz Doppler transducer, which provides real-time data on the arterial deformations. The patient-specific cyclic loading on the PSAM is extrapolated from the strain energy function developed using historical stress–strain relationships. Multivariant factors are proposed to locate points of arterial weakening that precede rupture. Biaxial tensile tests are used to calculate the material properties of the artery wall, enabling the observation of the time-dependent material response in wall rupture formation. In this way, correlations between the wall deformation and tissue failure mode can predict the aneurysm’s propensity to rupture. This method can be embedded within the ultrasound measures used to diagnose potential AAA ruptures.

## 1. Introduction

Abdominal aortic aneurysm (AAA) is a common form of cardiovascular disease for which early diagnosis is critical. Progression of the disease can lead to aneurysmal rupture, which has a high mortality rate due to catastrophic blood loss [1,2,3,4,5,6,7]. In an AAA, the descending aorta becomes enlarged, with the enlarged segment sometimes extending within the abdomen up to the bifurcation region. In clinical studies, the aneurysm size is a crucial determinant of rupture risk and the morbidity and mortality associated with AAA [8,9,10,11]. The risk of aneurysmal rupture is affected by a number of patient-specific rheological factors which should be considered in the surgical intervention decision [8]. Numerical methods that researchers have proposed include, among other things, the Finite Element Analysis Rupture Index (FEARI) [7], Rupture Potential Index (RPI) [8,10], and Severity Parameter (SP) [12,13]. However, practical validation of these methods is still required. Clinically, it is indicated that an aneurysm diameter of 5.5 cm requires full patient monitoring and potential surgery to repair the aneurysm [14,15]. However, when the patient is not at risk of rupture, early surgical intervention is costly and exposes the patient to risk. Therefore, to better identify the risk of rupture, patient-specific biomedical factors should be considered in addition to aneurysm size, and these factors should ideally be collected non-invasively.

Computational Fluid Dynamics (CFD) analysis enables sophisticated models of realistic arterial wall deformations to be created to characterize arterial deformations and blood flow characteristics. Our model employs CFD to develop a model to predict AAA rupture. Changes to the morphology of the arterial wall, including the presence of an aneurysm, can be captured via ultrasound. Accurate measurement of the blood velocity vector requires a non-perpendicular sonification angle. Therefore, inconsistencies in the sonification angle may result in incorrect values for determining the velocity [16]. The literature suggests using Womersley or Poiseuille methods when considering the velocity distribution for the blood flow contours based on the maximum or average velocity [17]. Patient-specific wall deformations and their thickness heterogeneity are used in wall estimation to predict potential rupture localization [18].

Arterial wall integrity is determined by critical tissue elements that comprise the wall structure, principally elastin, as the dominant element, as well as collagen and smooth-muscle cells. Kleinstreuer and Zhonghua [19] identified that the collagen-to-elastin ratio is the critical determinant of the aortic wall structure. This was experimentally demonstrated on the stress–strain curve for different AAA material compositions [20]; changes to the composition, such as an increased ratio of collagen to elastic, altered the wall mechanics, increasing arterial wall stiffness and reducing its tensile strength. Proteolytic enzymes affected the collagen in the region of the aneurysmal wall deformation where localized inflammatory responses of the arterial wall were associated with the enzyme content during collagen degradation tests [21].

This paper describes developing a model to predict aneurysm rupture to identify whether surgery would be recommended. The novelty of this method is that it is instantaneous and patient-specific, based on non-invasively captured MRI or ultrasound data. CFD models implemented in a viscoelastic formulation will be used to determine the critical stresses and strains leading to rupture. The viscoelastic models will also help to assess the time required to rupture. Hopefully, this approach will trigger new thoughts about using computational techniques to help prevent unnecessary and costly early elective repair surgery.

This paper uses ultrasound data, gathered non-invasively, to determine the instantaneous aneurysm geometry to be used in the diagnosis process. Based on these images, a PSAM for each patient is developed, with the specific pressure wave determined using computational modeling. The strain energy is obtained to identify the relationship between the strain at high energy points and the rupture time. Correlation between the different strain energies and time are evaluated, and affected parameters can therefore be interrelated using an estimated cyclic loading calculation.

## 2. Materials and Methods

**Figure 1:**
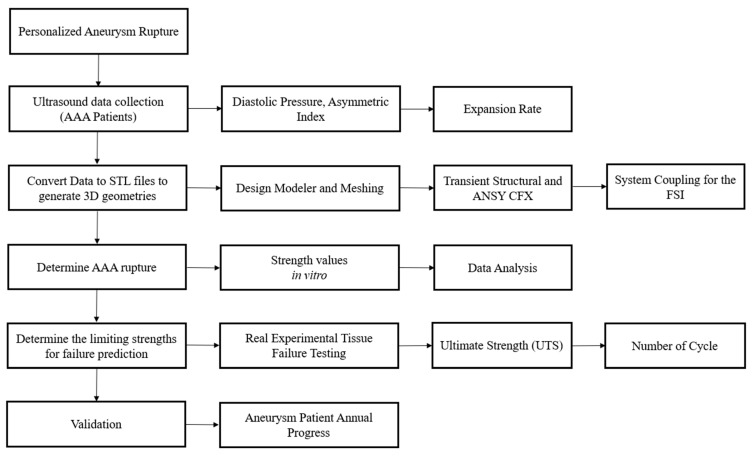
Flow chart for the methodology used.

The proposed methodology was based on four steps: (i) Ultrasound data were collected from AAA patients. (ii) These data were converted to geometrical CFD models. (iii) Based on previous in vitro AAA testing, rupture strength values were determined and used. (iv) Limiting strengths for failure prediction were determined. The flowchart for the current investigation is presented in Figure 1, to show the process used to conduct the experimental and computational work. 

### 2.1. Geometry Data Collection

**Figure 2:**
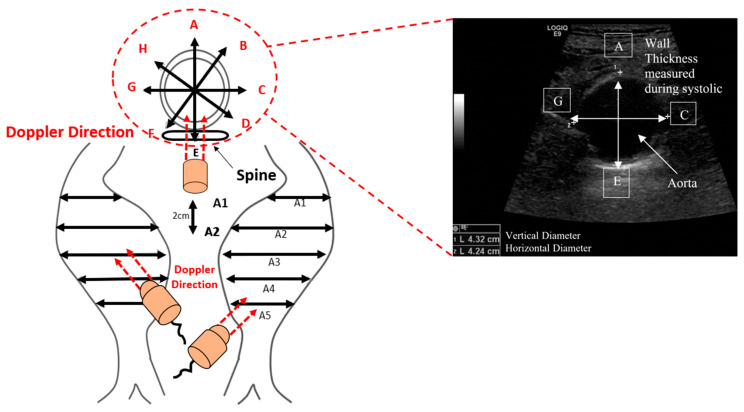
Process used to collect the 2D image’s data from the ultrasound. A1–A5 are cross-sectional segments used to render ultrasound images, with the black arrows illustrating the artery’s diameter at each cross-section. The red dashed arrows reflect the orientation of the doppler device. The letters in upper-case (A–H) reflect diameter boundaries for the artery, having different orientations.

Ethical approval was obtained according to the Manukau District Health Board-CMDHB1404, Auckland, New Zealand, regulations, and all patients’ consent was obtained accordingly. These patients visited the clinic regularly to monitor their AAA and general health conditions. Ultrasound data were collected from the patient’s records, from which demographic evidence and the geometry of the deformation in the patient’s artery were obtained. The Patient-Specific Aneurysm Rupture Prediction (P-SARP) approach was employed to obtain images at approximately 2 cm depth, from the region of the renal arteries to the bifurcation zone (see Figure 2). 

Ultrasound images were acquired using a General Electric (GE) ultrasound machine equipped with software (version 3.1.2). A curvilinear probe (C1-5) and 9L (Linear) probe were used for enhancing the deep tissue screening. The data were collected following the standard clinical assessment procedures approved by the AUT University Ethics Committee (AUTEC-12/273), with a vascular nurse, under the supervision of Dr Giri Mahadevan (CMDHB Vascular Surgeon), operating the device. Patients participated in the study voluntarily, with patient consent obtained according to the ethical approval obtained as described above.

The following steps were followed to collect the ultrasound images and data: (i) a scale sticker was attached to the patient’s stomach to conduct measurements at different segments from A1 to A5 based on the sections using an appropriate sonification angle, as shown in Figure 2; (ii) the spinal cord of the AAA patient was located at point E, as shown in Figure 2; (iii) the vascular nurse (collecting the data) ensured determination of the required dimensions with a cross-sectional area at a 45° angle (lumen wall and thickness) based on the nodes (A–C) and (E to G); and (iv) each segment was constructed by separating the lumen and artery wall, as shown in Figure 2. 

### 2.2. CFD Models

**Figure 3:**
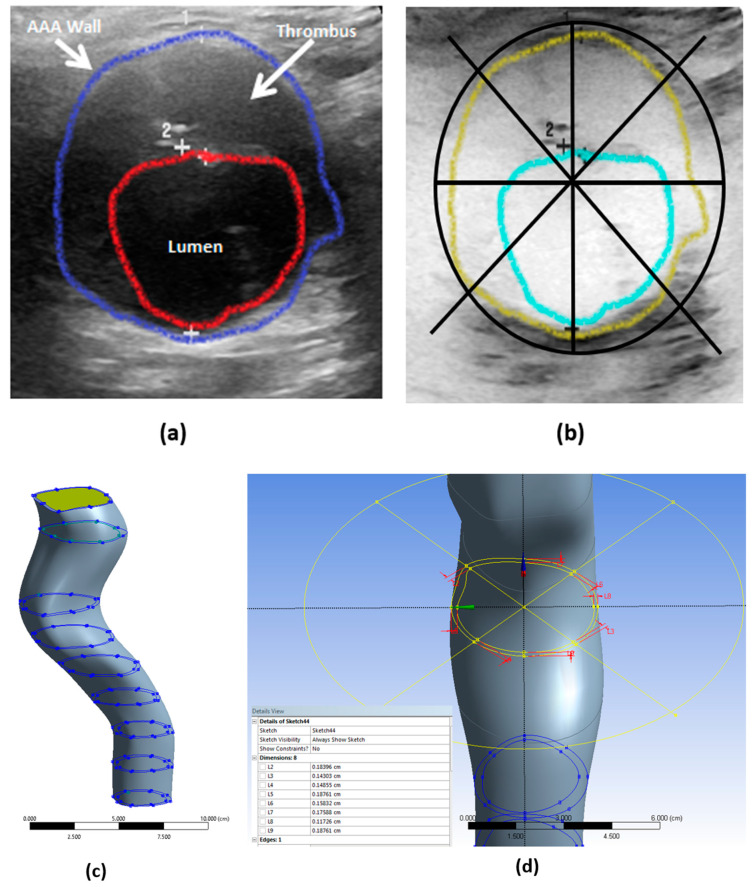
Patient-Specific Aneurysm Rupture Prediction (P-SARP) method. (**a**) Dimensions using ultrasound-segmented images template. (**b**) Arterial wall thickness measurements at a 45° angle, (**c**) Conversion of the 2D segments to 3D STL geometry, and (**d**) the dimensions to create each segment.

Using P-SARP data, ANSYS software was used to build a 3D model for each patient, Figure 3c. The necessary information included dimensions, velocity, thickness, and pulse wave velocity, and an estimated average blood pressure measurement was provided using a Pulsecor CardioScope II from Pulsecor Ltd. (Auckland, New Zealand). Patient-specific data was employed to precisely capture the deformation characteristics of different segments of the aorta over time. These images included those obtained via the ultrasound’s visual recorder and stills from the ultrasound scan. Details of the CFD modeling are presented in the next section.

**Figure 4:**
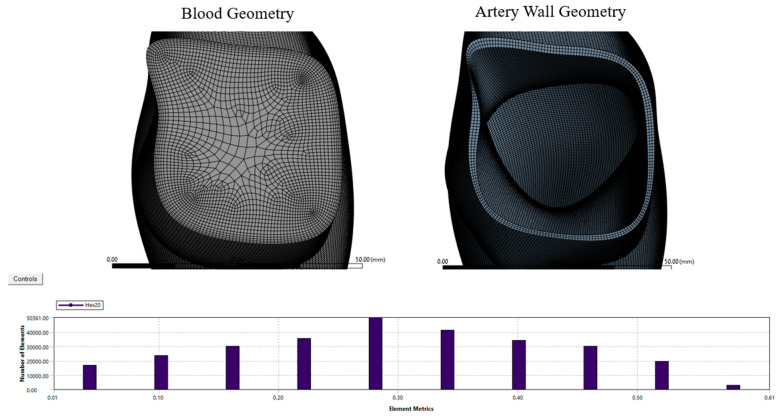
The mesh top view of the artery wall, the blood geometry, and the skewness chart showing the element metrics with the number of elements used in this study.

The element size for the blood geometry was set to 10.45 mm, with edge sizing set to 200 divisions using the sweep mesh method. For the artery wall, the mesh element size was set to 0.5 mm using two mapped faces and three as the internal number of divisions, using the sweep mesh method. The mesh quality for the artery wall was assessed to a skewness maximum value of 0.61 to achieve 1,419,506 nodes and 283,404 elements, as shown in Figure 4. The mesh method was set to use Sweep with a mapped face of two and with an internal number of divisions equal to three. Based on our previous studies [22,23,24], the mesh quality was acceptable, which was considered a good mesh for these types of non-uniform geometries.

The CFD model is established in the ANSYS- Workbench by putting the artery wall geometry under transient structural analysis and the blood flow geometry under ANSYS-CFX, with both being connected to System Coupling in order to perform the fluid–structure interaction (FSI) method. This process addresses the arterial wall deformation (in AAA) and its compliance to the pulsatile blood flow. The transient structural analysis assists in calculating the strain intensity and strain energy for each patient.

### 2.3. Tissue Stress–Strain

We obtained the stress–strain curves for the blood vessel wall, with permission, from the Nagoya Institute of Technology (Nagoya, Japan) Center for Fostering Young and Innovative Researchers [25]. The aneurysm tissues were obtained postmortem from six selected patients, as described in Table 1. These were tested, ex vivo, using a pressure-imposed test, which entailed a maximum pressure load of 4500 mmHg. As this is a preliminary investigation, it is assumed that the ex vivo tissue will not change its characteristics significantly. 

**Figure 5:**
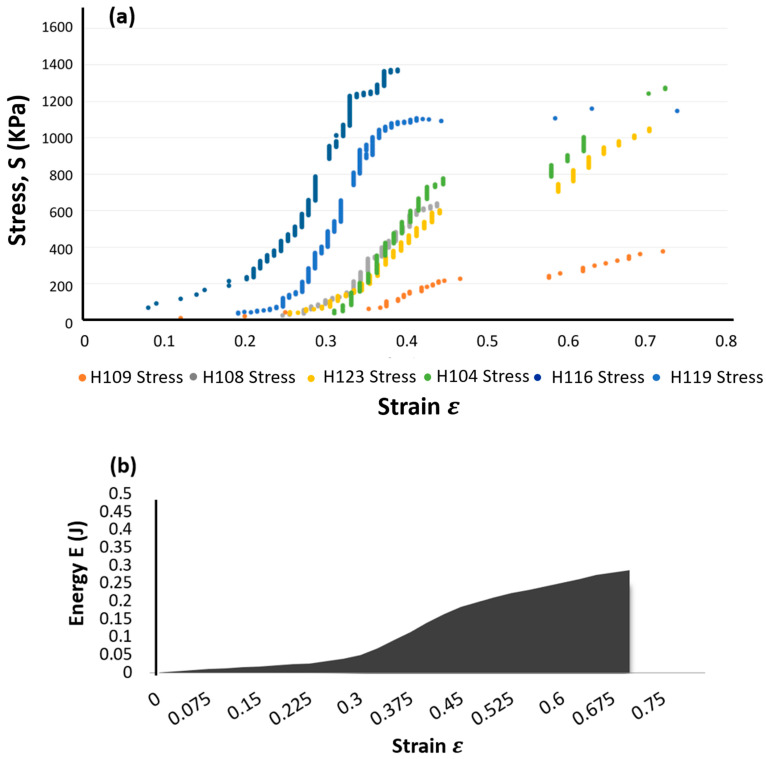
(**a**) Stress–strain curve for the ruptured specimens based on pressure-imposed testing. (**b**) Strain energy.

A specimen of a specific size was excised from the aneurysmal tissue and was tested using a device fabricated to measure the rupture properties. The experimental procedure consisted of: (i) the generation of air pressure via a compressor with an electro-pneumatic regulator; (ii) the gradual application of pressure, via a rubber balloon, in order to create a bulge in the specimen at the rate of 10 mmHg until either specimen rupture was achieved, or a maximum pressure of 4500 mmHg was reached; (iii) the measurement of the actual pressure applied to the specimen via the pressure transducer; and (iv) the generation of the tangent modulus average values of the stress–strain curves for the specimens, as shown in Figure 5a [25]. 

Four parameters that contribute to tissue failure were determined, namely, maximum stress (σ_max_), maximum strain (ε_max_), maximum energy (E_max_)—the area under the stress–strain curve, Figure 5b—and maximum strain energy per unit time (U_T_). The rate of the strain energy is determined by

(1) $$T=\int_{0}^{\varepsilon r}Sd\varepsilon$$

where *S* is the stress in kPa and εr is the incipient fracture strain.

Based on a previous study conducted by one of the co-authors, proximal specimens had a maximum average ultimate stress at rupture of 1.8 MPa, whereas distal specimens achieved a maximum average ultimate stress at rupture of 2.3 MPa [25]. 

We used the stress–strain curves obtained from the ruptured specimens to estimate the total energy at the center and edge locations. Due to the fact that thoracic aortic aneurysms (TAAs) which ruptured at the center differ from those ruptured at the edge, these were analyzed separately because the tensile strength calculated for the TAAs which ruptured at the edge might be smaller than the true tensile strength of the specimens. Figure 5a depicts this, as well as the total strain energy required under the specimens’ plotted curves. In this study, we determine the location where the strain energy is most significant per heartbeat, then from the pressure-imposed testing, the required strain energy to cause rupture and the approximate rupturing heartbeat were determined.

From the above experimental data, we can determine the point of the maximum load causing localized material deformation and then rupture. The ultimate strength (U_T_), or the material’s toughness model, can be used in calculating the total energy or the area under the stress–strain curve up to rupture. 

### 2.4. Failure Prediction

Using the P-SARP method for standardization, we defined the arterial wall according to the measured geometry. An accurate representation of the mechanical features of the arterial wall is critical for the appropriate modeling of an aneurysm. We must also accurately characterize the hemodynamical properties of the blood flow inside the vessel wall. Further, the complexity added by pulsatile flow must be considered, as some wall deformation is a natural part of blood flow in healthy vessels. In contrast, wall deformation becomes problematic in unhealthy (aneurysmal) vessels. Therefore, we can identify the contributing factors to aneurysm rupture. To set up the boundary condition, we set the central blood pressure waveform and pulse wave velocities at the inlet and outlet as fixed for each patient. The general fluid properties were set according to published data [26]. Simulation with ANSYS Fluid–Structure Interaction (FSI) was used to evaluate the 3D hemodynamic stresses in the blood vessel wall models. By employing Fluid–Structure Interaction (FSI), the outcomes are generated based on the applied fluid pressure and the velocity over the designated FSI surface within the model. The CFX solver, in turn, solves a set of algebraic multi-grid equations to yield robust results, particularly suitable for complex flow fields, as illustrated in Equation (2):

(2) $$\frac{d}{{dt}_{v}}\int_{\left(t\right)}^{}\rho d=\int_{v}^{}\frac{\partial \varphi }{\partial t}d\rho +\int_{s}^{}\varphi W_{j}{dn}_{j}$$

where ρ is the density of the blood, v is the volume of the blood flow, s is the surface of interface of the solid and fluid, $W_{j}$ is the blood velocity based on the control volume, and ${dn}_{j}$ is the cartesian component. 

The boundary conditions for each of the solid and fluid domains were set as follows, with the FSI interface between the solid and fluid domains: 

The solid domain, shown in Figure 5a, assumes the artery wall has a flexible geometry that responds to the wave propagations. After fixing both ends (zero displacements at each end), the body can have the possibility of radial displacement.

**Figure 6:**
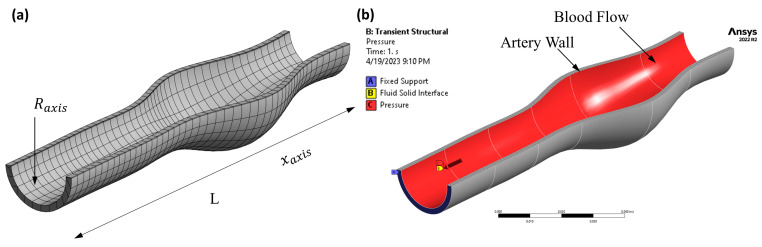
(**a**) The artery mesh model (**b**) The FSI interface (highlighted in red).

The fluid domain, shown in Figure 6b, is assumed to be an incompressible Newtonian fluid with a dynamic viscosity of 3.5 × 10^−3^ and a density of 1050 kg/m^3^, as recommended by the literature [22,23,24]. The fluid domains were fixed at the inlet and outlet. 

This study’s input and output boundary condition data were set using clinical data on pressure waveforms as a function of time, obtained through invasive measurements from patients undergoing aneurysm assessments at the Vascular Surgery Department within the Manukau Super Clinic, under the ethical approval number 1404. The equation is determined by employing a Fourier transform formulation, which is based on a consistent waveform derived from imaging data used in our previous study [26].

**Figure 7:**
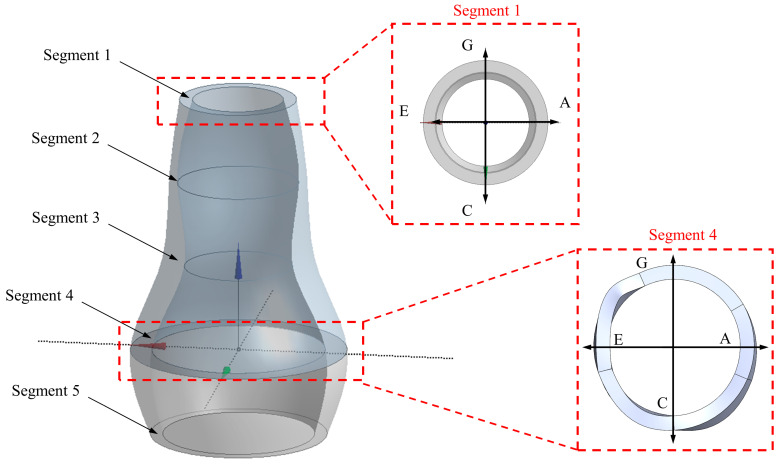
The construction of the aneurysm geometry using the P-SARP protocol showing multiple thickness variations.

As a PSAM, the input and output in this setup use a selected patient’s blood pressure waveforms obtained from patients undergoing an aneurysm assessment (an invasive procedure), with consent provided under ethical protocols (ethics number 1404). A Fourier transform function governs the input and output wave equations, with the waveform cycle consistent with the imaging data. The model was simplified without changing any aspects of the geometry to retain the geometry consistent with the patient’s data. Figure 7 shows that the model contains five to six segments with four circumferential points. These simplified models allow us to investigate how the disease progresses in a given patient. 

## 3. Results and Discussion

**Figure 8:**
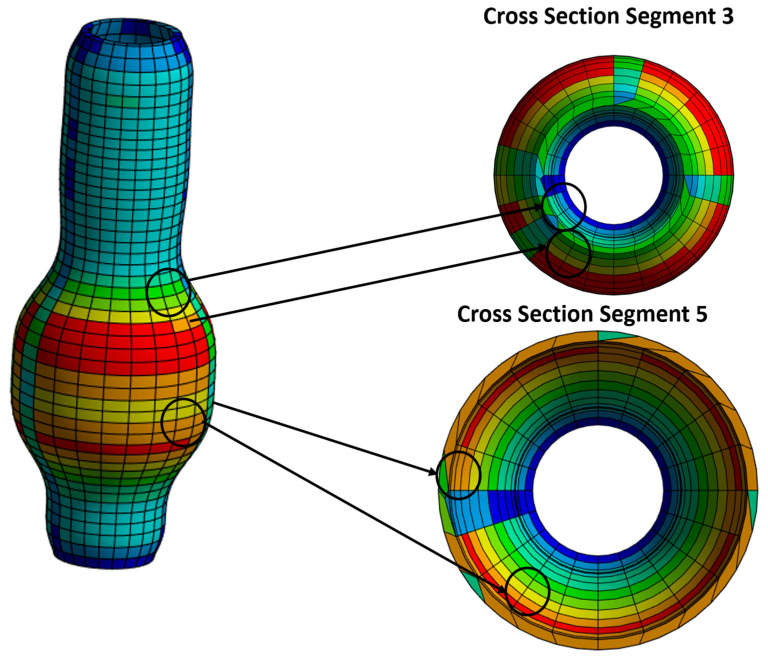
Segments 3 and 5, showing high strain energy as obtained from transient structure analysis.

A simulation analysis was performed, subjecting the abdominal aorta structure to stresses, strain deformation, and energy, to examine the aneurysm’s behavior under different parameters and at different stages, Figure 8. The structure’s geometry was varied to determine the deflection variation given the model’s shape and material properties. This also enables the examination of any particular thickness and enlarged diameter locations in the model. The simulation result was compared to those of different patients to identify variations between the simulated aneurysm’s geometry and the actual location of the patient’s aorta. Further analysis and comparison of these particular parameters will identify the characteristics leading to rupture.

(3) $$\left(U_{T}\right)=\frac{Total\;Energy\;required\;to\;rupture\;(U_{f})}{Time}$$

**Figure 9:**
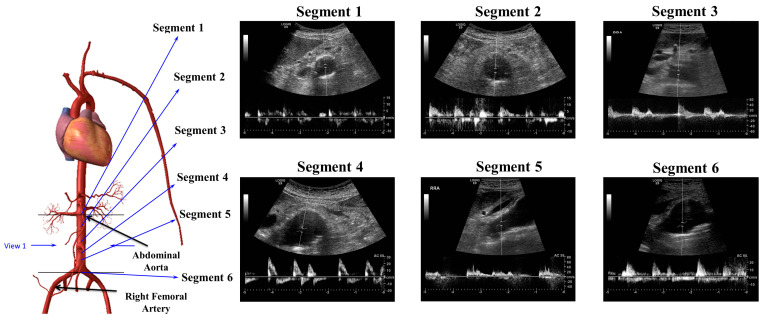
Stylized model of abdominal aorta and ultrasound data at different segments including circumference and flow velocities for each segment.

CFD analysis was used to calculate the strain energy at the zone of high deformation. To find the location of the potential rupture, further analysis of the lumen diameter and specific circumferential strain value of the affected segment must be performed. Figure 9 presents ultrasound data obtained from a particular patient before image reconstruction. The variations of the aneurysmal arterial wall can be more accurately modeled using these instantaneous images. 

The results from the numerical model were used to obtain a measure of the strain energy per unit time using ANSYS. This value was calculated experimentally from the tissue failure specimens, where the final ultimate load was identified. The strain energy per unit time ($U_{T}$) was calculated according to Equation (3) below, which enables the selection of an acceptable parameter to be embedded in the model and correlates the model’s results to the real specimens for a given diameter and thickness. This allows for the potential to non-invasively observe rupture-inducing factors. 

During the specimen analysis, the failure parameter for the sample was determined based on the position and dimensions during testing, where an average strain energy of 111.14 J was calculated across the six specimens (based on the amount of displacement in the stress–strain testing). The more elastic the tissue, the more significant the displacement which can be absorbed by the tissue until artery failure (where all tissues across the specimen wall tear) occurs. Consequently, an artery wall can fail earlier than when suggested by the visualized images.

Table 2 describes the combination of determinants for the correlation between aneurysmal diameter and rupture for each PSAM. This can deliver the ultrasound assessment of aneurysm patients; based on the strain energy results in the model, the possible location, current thickness, and time estimation can be determined. Given the results from the simulation, this calculation gives an estimated time to rupture and, therefore, whether intervention can be indicated. 

Under a continuous pulsatile load, the material undergoes ongoing deformation in response to the heartbeat-generated pressure. Once it attains a consistent level of strain, we record the time it takes to reach this point. This time factor corresponds to the tissue’s capacity to resist further deformation or fatigue after surpassing its yield strength. This method enables the determination of the energy consumption beyond the elastic region based on the stress–strain data.

Using correlations between our model’s strain energy rates and those rates identified during specimen testing enables an estimation of the time to rupture or the number of heartbeats until the tissue ruptures. However, the heightened wall tension during aneurysm growth and the wall strength factor may lead to sudden rupture [27]. This wall strength factor cannot be found easily via in vivo examination; therefore, we must examine ex vivo specimens to develop some insight into the tissue’s behavior under different conditions. This is particularly necessary given the resource intensiveness for in vivo wall stress estimation [28]. 

In this study, we can use the information provided by the heart rate to examine the motion of the wall tissue given different pulsatile pressures and cyclic loadings. The relationship between the pulse pressure and wall size changes can be identified based on the number of cycles. For aneurysms examined in vivo, spontaneous changes to the blood pressure can occur due to inconsistencies in the heart rate cycle, which alter the internal forces acting on the aneurysm’s wall. Consequently, the number of cycles can be estimated by measuring inconsistent heart rates in a given period and evaluating the percentage variation. Gilpin [29] conducted a cyclic fatigue test to find high and low ultimate stress percentages in test profiles across a number of cycles. This method is explored in the following subsection.

### 3.1. Strain Energy to Stress-Cycle (S-N) Curve

**Figure 10:**
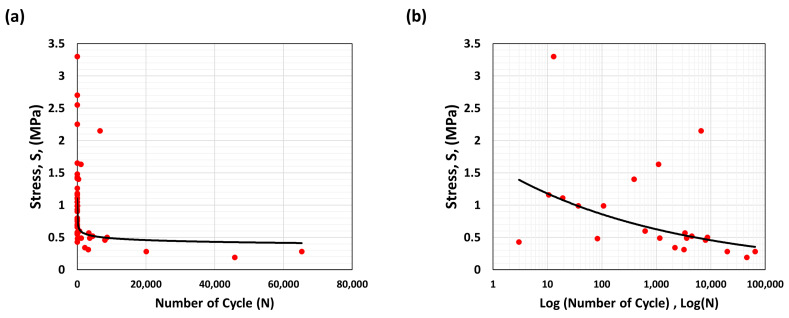
The S-N curves obtained for cyclic loading in specimens [18] showing (**a**) the number of cycles (N), and (**b**) Log (N).

The Stress-Cycle (S-N) curve can be plotted using data gathered from the cyclic loading experiment based on an estimation of pressure-cycle loading. This S-N curve can be used to predict tissue failure by elucidating the potential failure points and could be used to estimate the behavior of in vivo tissue, as shown in Figure 10. Blood pressure, which affects vessel dilation, can represent the degree of local stress on the tissue. Considering these stress factors, a broad estimate of the number of cycles to rupture can be found under cyclic loading. While the measure may be an inexact estimate, this is preferred to no estimate, leading to aneurysmal rupture or potentially unnecessary surgical intervention [29]. One of the limitations arises from the challenge of extracting the tangent elastic modulus directly from clinical in vivo data. Nevertheless, it is feasible to acquire the yielding parameter, specifically the yield of the wall material, from clinical data. Additionally, this parameter exhibits a noteworthy correlation with the maximum stress, also known as the ultimate stress, observed across all specimens.

The S-N curve also helps provide valuable information about the rupture, as its shape reflects thickness effects, where higher stress or energy strain on the tissue wall, irrespective of aneurysm size, can impact the normal cyclic load. 

### 3.2. Analysis of the Stress Life Cycle (S-N Curve)

As noted earlier, the high elasticity of tissue affects the time to rupture. For tissue with higher elasticity, the stress correlation coefficient has a limited value (indicating a weaker relationship). In the experimental tests, there was limited damage accumulation due to fatigue in such aneurysm tissues. In the S-N curves derived for these tissues, it can be observed that the cyclic loading is beneath the material’s ultimate strength. Specimens subjected to higher stresses before they experienced failure typically had higher stretch ratios than other specimens, which provides some experimental evidence for why aneurysm rupture does not always occur at the maximum diameter and, consequently, why the diameter of the aneurysm is not always a critical indicator of rupture likelihood. Histological samples generally reveal that damage due to fatigue is cumulative, with the time vs. position curve having a positive slope, indicating material failure [29]. Given the correlation identified between the maximum load stresses, the strain energy, and the number of cycles, these can be used as a basis for the estimated failure point for the tissue, as given by the number of cycles to failure.

**Figure 11:**
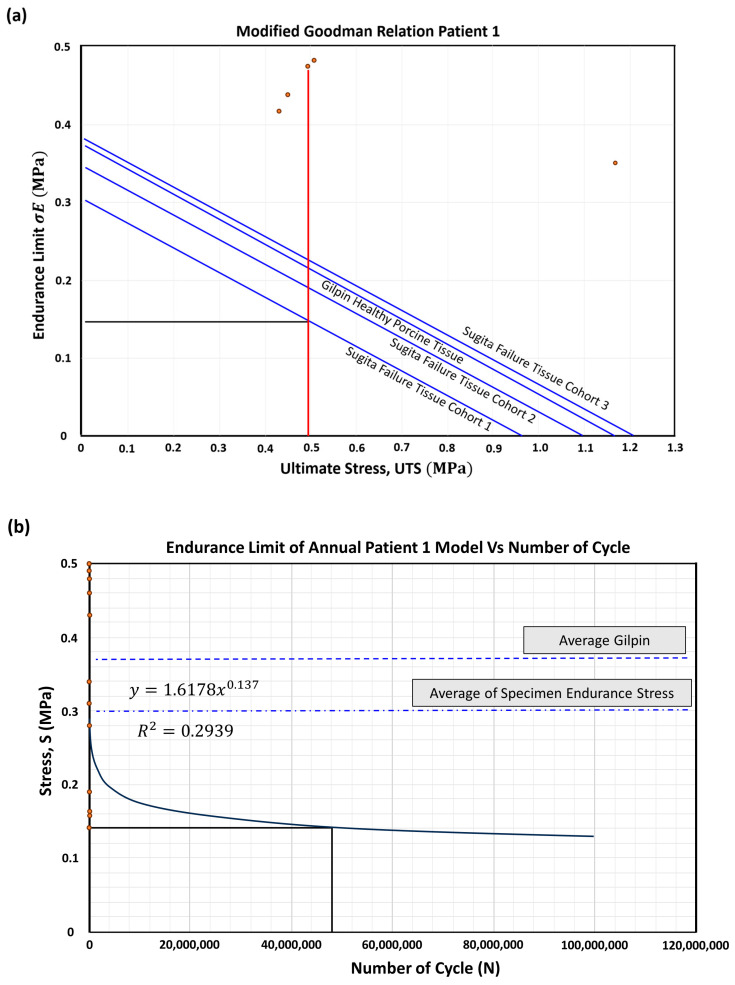
(**a**) Goodman relation for PSAM-failure group specimen and (**b**) number of cycles for PSAM-failure group specimen.

Figure 11 plots the S-N curve against the fatigue graph to calculate the endurance stress for each PSAM model. From the stress maxima and minima obtained in the model, a linear graph can be constructed, as shown in Figure 11. It is essential to distinguish between the specimens with a range of aneurysmal diameters and tissue wall properties and the models. Hence, we plot an individual line before determining the number of cycles. 

Figure 11a demonstrates how we adapted Goodman’s theory to patient 1 in order to establish the Sugita Failure Tissue within Cohort 1. This was carried out to match the rheological properties and estimate the endurance limit of the artery wall, relying on the ultimate stress. Subsequently, Figure 11b was constructed to illustrate the yearly progression of patient 1’s aneurysm, leveraging Manukau data, which tracks the historical development of the aneurysm. These data were used to derive stress information and develop an equation based on the number of cycles for the PSAM-failure group specimen of patient 1. Consequently, we obtained strain energy values to identify the relationship between the strain at high-energy points and the time to rupture for the patient.

We determined the strain energy to rupture through cyclic loading for each specimen. Further, the maximum strain energy at a specific heart rate was obtained via Computational Fluid Dynamics (CFD) modelling using the PSAM technique. To validate this, we approximated the heart rate at rupture by dividing the first point by the second point, resulting in Figure 11a, depicting the S-N curve for the PSAM-failure group specimen. Figure 11b shows the number of cycles for the PSAM-failure group specimen. Table 3 presents the strain energy data for each specimen. 

Further validation will be possible as part of future work, as patients’ progress is monitored by their physician; however, these data are not available under the current ethical approval framework obtained for this study.

## 4. Conclusions

Several factors influence the time to an aneurysmal rupture, whereas most clinicians have access to one crucial factor, the diameter of the aneurysm, non-invasively via ultrasound techniques. When subjecting tissue specimens to pressure tests, the time to rupture differs depending on wall thickness and elasticity, which are difficult to measure non-invasively. An approximate rupture time can be calculated considering these patient-specific factors and using numerical simulation. The PSAM model can identify the location of the area vulnerable to rupture and estimate the extent of the cyclic loading to which it can be subjected. Using strain energy, the PSAM model determines the distribution of stresses, in vivo, and the abdominal aorta wall’s material strength, enabling a prediction of the approximate time to aneurysm rupture. The vulnerability of the patient to aneurysmal rupture can be found by relating the model to the instantaneous high-strain energy found via readily available ultrasound imaging techniques. This relies on the assumption that the ex vivo tissue’s characteristics have not changed significantly.

## Figures and Tables

**Table 1:** Demographic and dimensional data for the six aneurysm specimens [25].

Specimen	Origin	Sex	Age	Position	Diameter (cm)	Thickness (cm)
H0104A (P1)	Annulo Aortic Ectasia	F	74	Descending	5	0.27
H0123 (13)	True Aneurysm	M	63	Descending	6.5	0.41
H0108 (P3)	True Aneurysm	M	68	Arch	5	0.33
H0109 (P4)	Aortic Dissection	M	70	Descending	6.2	0.34
H0116A (P10)	True Aneurysm	M	73	Arch	6.5	0.38
H0119A (P12)	True Aneurysm	M	67	Arch	6.2	0.43

**Table 2:** Classification of patients according to their aneurysm diameter.

Cohort	Diameter (cm)	In-Range Excised Specimen from Sugita Experimental Data	Average Average per Unit Time $\left(U_{T}\right)$	Relative Patient Data from Ultrasound Image	Average Ultimate Strength, UTS (MPa)	Total Energy to Failure (J)
1	4.0–5.4	H104 (5.0) H108 (5.0)	0.41	P1, 3, 4, 5, 7, 9, 11 & 12	0.96	76.64
2	5.5–6.4	H119 (6.2)	0.37	P2, 6, 8 &10	1.15	140.75
3	≥6.5	H116 (6.5) H123 (6.5)	0.49	P13	1.21	156.47

**Table 3:** The energy strain data for each specimen.

	H104	H108	H119	H116	H123
Energy Strain $U_{T}\;(J)$	0.45	0.38	1.22	0.43	0.54

## Data Availability

Not applicable.
